# Pattern of Pediatric Dermatoses in a Tertiary Care Hospital of Western Nepal

**DOI:** 10.1155/2016/6306404

**Published:** 2016-05-09

**Authors:** Yogesh Poudyal, Annu Ranjit, Santosh Pathak, Nagendra Chaudhary

**Affiliations:** ^1^Department of Dermatology, Universal College of Medical Sciences, Bhairahawa 32900, Nepal; ^2^Department of Pediatrics, Universal College of Medical Sciences, Bhairahawa 32900, Nepal

## Abstract

Pediatric dermatoses are one of the most common presentations in a dermatology clinic and reflect the health and hygiene status of children. The incidence and severity of these skin lesions are influenced by geographical area, seasonal and cultural factors, and socioeconomic status. This study was done to show the prevalence of different pediatric dermatoses in a tertiary care hospital of Western Nepal. Chart reviews of children aged one day to 17 years, presenting to Universal Medical College Teaching Hospital, Nepal, from 1 September 2014 to 30 august 2015, were done. Descriptive analysis and two-sided chi-square test were done. Among 23992 patients visiting the dermatology outpatient department (OPD), 5398 (22.5%) were of pediatric age groups (male/female: 1.2/1); most of them belonged to young teens and teenagers (*n* = 3308; 61.3%). Three most common dermatoses were fungal infections (18.5%), eczema (14.4%), and acne (10.1%). Fungal infections (*n* = 653; 65.4%) and acne (*n* = 284; 51.9%) were common in males, whereas eczema (*n* = 402; 51.7%) was more common in females. Fungal infection (*P* < 0.001), eczema (*P* < 0.001), pigmentation disorders (*P* < 0.001), and acne (*P* < 0.01) were significantly more during summer, while scabies was more in winter (*P* < 0.001). Dermatophytosis, pyoderma, and warts comprised frequently occurring fungal, bacterial, and viral infections, respectively.

## 1. Introduction

Pediatric dermatology is an important branch of dermatology that deals with the diagnosis, treatment, and prevention of skin diseases occurring in infancy, childhood, and adolescence [1]. Skin diseases are common in children, the incidence being 9–37% all over the world with almost 13.4% in Nepal [2, 3].

Dealing with a child with skin disorders requires special skills as they differ in clinical presentation, treatment, and prognosis when compared with adult dermatosis [3]. It has been observed that a pediatrician encounters almost 30% of all his outpatient children having dermatological problems while a dermatologist encounters about 30% of children visiting the outpatient department [4].

Studies of pediatric population suffering from skin diseases can play an important role in public health and policy making [5]. Many factors like geographical area, climate, season, and socioeconomic status influence the pattern of skin diseases in children. Various studies conducted in different parts of the world shows different patterns of skin diseases in children.

This study aims to find different patterns of skin diseases in pediatric population along with the seasonal variation in western part of Nepal.

## 2. Materials and Methods

This was a retrospective study conducted in department of dermatology at Universal College of Medical Sciences-Teaching Hospital (UCMS-TH), a tertiary care referral hospital situated in Rupandehi District of western region of Nepal. This region is a tropical zone with humid summers. More than 90% of the patients visiting this hospital are from Terai region of Nepal (a plain area in Nepal nearby Uttar Pradesh state of India). The data of children, aged from one day to 17 years, between 1 September 2014 and 31 August 2015, were collected from the record section of the hospital. All new and old cases were included in the study. All the diagnoses were made by dermatologists based on detailed history, clinical presentation, and appropriate investigations. The age group of children included in the study was divided into 0-1 year (infants), 1–5 years (toddlers and preschoolers), 6–11 years (middle childhood), and 12–17 years (young teens and teenagers). The diagnoses were grouped into 13 categories; any diagnosis which did not fit into these categories was included in other categories. The 13 categories included were acne, bacterial infections, bite reactions, drug reactions, eczema, fungal infections, hair disorders, naevus, papulosquamous diseases, pigmentary diseases, scabies, urticaria, and viral infections. Frequencies of diseases according to months and gender were recorded. Data was entered and tabulated in MS-excel sheet and descriptive analysis using variables (mean, percentage, and proportion) was done. Analytical analysis was done by calculating chi-square (*χ* ^2^) to compare different dermatoses with seasonal variation. *P* value of less than 0.05 was considered statistically significant.

## 3. Results

Out of total 23992 patients visiting the dermatology outpatient department (OPD), 5398 (22.5%) were of pediatric age groups. The three most common dermatoses were fungal infections (18.5%), eczema (14.4%), and acne (10.1%). Most frequently presenting age group was young teens and teenagers (61.3%). Eczema was most frequently presenting dermatosis followed by scabies in infants, toddlers, and preschoolers. In middle childhood, the commonest dermatosis was eczema followed by fungal infection, whereas fungal infection was commonest followed by acne in young teens and teenagers. The distribution of different dermatoses according to age groups is shown in Table 1. Young teens and teenagers were most frequently presenting age groups.

Male-female ratio was 1.2 : 1 as depicted in Table 2. Among the 3 common dermatoses, fungal infections (653; 65.4%) and acne (284; 51.9%) were common in males, whereas eczema (402; 51.7%) was more common in females. Majority of female children had pigment and papulosquamous disorders, hair disorders, and nevus, whereas large number of male children presented with bacterial infection, bite reaction, drug reaction, urticaria, and scabies.

The maximum number of dermatoses was seen in summer and autumn (fall), whereas winter had the least number. Winter includes the month of December, January, and February; spring includes March, April, and May. Summer includes June, July, and August, whereas fall includes September, October, and November. On comparing the 5 common dermatoses, 4 dermatoses, fungal infection (*χ* ^2^ = 26.0828; *P* < 0.000009), eczema (*χ* ^2^ = 21.9486; *P* < 0.000067), pigmentation disorders (*χ* ^2^ = 0.000633; *P* < 0.000633), and acne (*χ* ^2^ = 0.010228; *P* < 0.010228), were significantly more during summer, spring, and autumn seasons, while the number of scabies cases was more in winter season (*χ* ^2^ = 0.000586; *P* < 0.000586) statistically. The seasonal distribution of the five common dermatoses is shown in Table 3. Comparison of five common dermatoses with the seasons showed a strongly significant effect on occurrence of common dermatoses statistically (*P* < 0.05) (Table 3).

Fungal infections (75.2% of all infectious dermatoses) were most common infectious dermatoses followed by bacterial (13.7%) and viral infections (11.1%), respectively. Dermatophytosis was the most common fungal infection seen in our study followed by pityriasis versicolor and tinea capitis, respectively. The most common bacterial infection was pyoderma, whereas warts (94; 64.9%) were the commonest viral infections (Table 4). Total infectious dermatosis was 1564 (29%).

## 4. Discussion

There is paucity of epidemiological data in pediatric dermatoses. This retrospective study was done to show the prevalence of different pediatric dermatoses in a tertiary care hospital of Western Nepal and to look on the seasonal trends along with the pattern of its occurrence in different pediatric age groups.

It has been noticed that pediatric dermatoses vary from one country to another, one state to another, and even one society to another. Prevalence of pediatric dermatosis in our study was more than the previous studies conducted in the country [6, 7]. Another study done in the nation showed the prevalence of pediatric dermatoses similar to our study [3]. One study conducted in the hilly region of Nepal even showed prevalence of pediatric dermatoses even higher than our study [8]. This variation may be due to different cultural practices and difference in environment and socioeconomic status.

There was variation in prevalence of dermatosis among the age groups in our study. Fungal infection was the most common dermatosis overall in our study. Eczema was more common in the early age groups, whereas fungal infections were more common among later age groups in our study. A study from Turkey showed eczema as the commonest skin disorder in early age groups similar to our study whereas acne was more prevalent in adolescents [9]. Acne was second to fungal infections in our study among the adolescents. This shows that pediatric dermatosis shows variation among the different age groups within the pediatric population.

The frequency of male children (54.7%) was more common than female children. Eczema was more common in female showing similarity with study done by Wenk and Itin [10] and Muzaffar [11]. The number of patients visiting the dermatology OPD in age group 0-1 year was less compared to other age groups. The decrease in prevalence of infants visiting dermatology OPD may be due to parents visiting the pediatrician. The seasonal occurrence of different dermatoses were not much different except in case of scabies where the number of cases was more than double in winter as compared to summer. Banerjee et al. had similar findings in their study done recently [12]. The overall prevalence of scabies was 4.4% in our study whereas a study in Pakistan showed 5-fold more prevalence of scabies [13]. Poor hygiene in winters along with poor sanitation may be the cause of increased prevalence of scabies in developing countries.

Dermatophytosis was the most common dermatosis in our study. A similar type of study done by Shrestha et al. in the hilly region of the country showed eczema as the commonest dermatosis indicating the change in the pattern of dermatosis within the country according to regions and climate changes [3]. Infections and infestations are common in tropical regions of many Asian and African countries [14–16], whereas eczema and dermatitis are common in the western countries [17]. This suggests that hot and humid climate of a developing nation are more prone to infectious dermatoses. Poor sanitation and low socioeconomic status also may be an important factor for increased prevalence of pediatric dermatoses.

One interesting finding regarding the infectious dermatosis of our study was increased prevalence of pediatric fungal dermatoses in contrast to other similar studies where bacterial infection was more common [14–16, 18, 19].

A study done by Sayal et al. also demonstrated fungal infection to be more than bacterial and viral infection [20]. This states the significant burden of fungal infection in this part of the country differing from other parts of the world. Wart was the commonest viral infection in our study as compared to other studies [11, 14].

## 5. Conclusion

Overall fungal infections, eczema, and acne were the three major pediatric dermatoses occurring most commonly in the young teens and teenager groups. Summer and autumn seasons had large number of cases while winters the least number. Comparison of the common dermatoses with the seasons showed a strongly significant effect on occurrence of common dermatoses.

The magnitude and distribution of skin disorders in children in developing nations like Nepal can help in identifying the disease burden. It further helps the government, policymakers, and different health related organizations to take appropriate measures in diagnosing, providing adequate treatment, and undertaking various preventive measures. Data on seasonal trends can further help us take appropriate steps for its management.

## Figures and Tables

**Table 1:** Distribution of dermatosis according to age group.

Dermatosis	0-1 year	1–5 years	6–11 years	12–17 years	Total
	*N* (%)	*N* (%)	*N* (%)	*N* (%)	*N* (%)
Acne	1 (0.50)	0 (0.0)	8 (6.0)	538 (16.3)	547 (10.1)
Bacterial infection	11 (5.50)	45 (6.8)	42 (3.2)	83 (2.5)	188 (3.5)
Bite reaction	14 (7.0)	45 (6.8)	42 (3.2)	51 (1.5)	152 (2.8)
Drug reaction	1 (0.50)	0 (0.0)	5 (0.4)	5 (0.1)	11 (0.2)
Eczema	37 (18.7)	122 (18.3)	211 (15.9)	408 (12.3)	778 (14.4)
Fungal infections	20 (10.1)	68 (10.2)	181 (13.6)	729 (22.0)	998 (18.5)
Hair disorders	0 (0.00)	4 (0.6)	44 (3.3)	44 (1.3)	92 (1.7)
Naevus	9 (4.50)	10 (1.5)	16 (1.2)	26 (7.9)	61 (1.1)
Others	64 (32.3)	211 (31.7)	360 (27.1)	821 (24.8)	1456 (27.0)
Papulosquamous disorders	1 (0.5)	6 (0.9)	26 (1.9)	54 (1.6)	87 (1.6)
Pigment disorders	6 (3.0)	42 (6.3)	168 (12.7)	284 (8.6)	500 (9.3)
Scabies	31 (15.7)	59 (8.9)	64 (4.8)	84 (2.5)	238 (4.4)
Urticaria	2 (1.0)	39 (5.9)	28 (2.1)	81 (2.4)	150 (2.8)
Viral infections	1 (0.5)	14 (2.1)	32 (2.4)	100 (3.0)	147 (2.7)

*Total*	*198*	*665*	*1327*	*3308*	*5398 *

**Table 2:** Gender distribution of different dermatoses.

Dermatosis	Male	Female	Total (*N*)
	*N* (%)	*N* (%)	
Acne	284 (51.9)	263 (48.1)	547
Bacterial infection	106 (56.4)	75 (43.6)	188
Bite reaction	79 (52.0)	73 (48)	152
Drug reaction	7 (63.7)	4 (36.3)	11
Eczema	376 (48.3)	402 (51.7)	778
Fungal infection	653 (65.4)	345 (34.6)	998
Hair disorders	44 (47.8)	48 (52.2)	92
Naevus	10 (16.4)	51 (83.6)	61
Others	791 (54.3)	665 (45.7)	1456
Papulosquamous disorders	42 (48.3)	45 (51.7)	87
Pigment disorders	217 (43.4)	283 (56.6)	500
Scabies	157 (66.0)	81 (34)	238
Urticaria	108 (72.0)	42 (28)	150
Viral infection	79 (53.7)	68 (46.3)	147

Total	2953	2445	5398

**Table 3:** Seasonal trend of five common dermatoses.

Diagnosis	Number (%)				Mean	*χ* ^2^	*P* value
	Spring	Summer	Autumn	Winter			
Fungal infection	288 (28.04)	235 (22.88)	303 (29.50)	201 (19.57)	256.8	26.0828	0.000009
Eczema	232 (29.82)	179 (23.01)	218 (28.02)	149 (19.15)	194.5	21.9486	0.000067
Pigmentation disorders	129 (25.80)	144 (28.80)	141 (28.20)	86 (17.20)	125.0	17.232	0.000633
Acne	115 (21.02)	151 (27.61)	161 (29.43)	120 (21.94)	136.8	11.2962	0.010228
Scabies	54 (22.69)	36 (15.13)	70 (29.41)	78 (32.77)	59.5	17.395	0.000586

**Table 4:** Pattern of infectious dermatosis (*N* = 1326).

Infections	Number (%)
Fungal infections (*N*: 998)	
Tinea capitis	65 (6.5)
Other dermatophytosis	773 (77.5)
Pityriasis versicolor	127 (12.7)
Candidiasis	33 (3.3)
Total (A)	998

Bacterial infections (*N*: 181)	
Pyoderma	156 (86.2)
Other	25 (13.8)
Total (B)	181

Viral infections (*N*: 147)	
Molluscum contagiosum	24 (16.3)
Wart	94 (64.9)
Other	29 (19.8)
Total (C)	147

*Grand total (A + B + C) *	*1326*

## References

[1] BenSaif G. A., AlShehab S. A. (2008). Pattern of childhood dermatoses at teaching hospital of Saudi Arabia. *International Journal of Health Sciences*.

[2] Hassan I., Ahmad K., Yaseen A. (2014). Pattern of pediatric dermatoses in Kashmir valley: a study from a tertiary care center. *IJDVL*.

[3] Shrestha R., Shrestha D., Dhakal A. K., Shakya A., Shah S. C., Shakya H. (2012). Spectrum of pediatric dermatoses in tertiary care center in Nepal. *Nepal Medical College Journal*.

[4] Thappa D. M. (2002). Common skin problems in children. *Indian Journal of Pediatrics*.

[5] Mostafa F. F., Hassan A. A., Soliman M. I., Nassar A., Deabes R. H. (2012). Prevalence of skin diseases among infants and children in Al Sharqia Governorate, Egypt. *Egyptian Dermatology Online Journal*.

[6] Neupane S., Pandey P. (2012). Spectrum of dermatoses among paediatric patients in a teaching hospital of Western Nepal. *Nepal Journal of Dermatology, Venereology & Leprology*.

[7] Kumar A., Mishra A., Devkota S., Sathian B., Koirala D. P., Paudel U. (2010). Pattern of pediatric skin disorders in tertiary care center in Western Nepal. *Nepal Journal of Dermatology, Venereology and Leprosy*.

[8] Shrestha D. P., Gurung D., Rosdahl I. (2013). Prevalence of skin diseases and impact on quality of life in hilly region of Nepal. *Journal of Institute of Medicine*.

[9] Gül Ü., Çakmak S. K., Gönül M., Kiliç A., Bilgili S. (2008). Pediatric skin disorders encountered in a dermatology outpatient clinic in Turkey. *Pediatric Dermatology*.

[10] Wenk C., Itin P. H. (2003). Epidemiology of pediatric dermatology and allergology in the Region of Aargau, Switzerland. *Pediatric Dermatology*.

[11] Muzaffar F. (2012). Pattern of skin diseases at The Children's Hospital, Lahore: comparison between 1996–1998 and 2011. *Journal of Pakistan Association of Dermatologists*.

[12] Banerjee S., Gangopadhyay D. N., Jana S., Chanda M. (2010). Seasonal variation in pediatric dermatoses. *Indian Journal of Dermatology*.

[13] Javed M., Jairamani C. (2006). Pediatric dermatology: an audit at Hamdard University Hospital, Karachi. *Journal of Pakistan Association of Dermatologists*.

[14] Al-Tawara M., Odeibat H., Al-Smadi R., Omeish I., Obaidat N. (2014). Patterns of skin diseases among pediatric patients attending the pediatric dermatological clinic at King Hussein Medical Center. *Journal of the Royal Medical Services*.

[15] Balai M., Khare A. K., Gupta L. K., Mittal A., Kuldeep C. M. (2012). Pattern of pediatric dermatoses in a tertiary care centre of South West Rajasthan. *Indian Journal of Dermatology*.

[16] Mostafa F. F., Hassan A. A. H., Soliman M. I., Nassar A., Deabes R. H. (2012). Prevalence of skin diseases among infants and children in Al Sharqia Governorate, Egypt. *Egyptian Dermatology Online Journal*.

[17] Del Pozzo-Magaña B. R., Lazo-Langner A., Gutiérrez-Castrellón P., Ruiz-Maldonado R. (2012). Common dermatoses in children referred to a specialized pediatric dermatology service in mexico: a comparative study between two decades. *ISRN Dermatology*.

[18] Vora R., Bodiwala N., Patel S., Krishna S. (2012). Prevalence of various dermatoses in school children of Anand district. *National Journal of Community Medicine*.

[19] Oyedeji O. A., Onayemi O., Oyedeji G. A. (2008). Prevalence and pattern of akin infections and infestations among primary school pupils in Ijesha land. *Nigerian Journal of Paediatrics*.

[20] Sayal S. K., Bal A. S., Gupta C. M. (1997). Pattern of skin diseases in paediatric age group and adolescents. *Indian Journal of Dermatology, Venereology and Leprology*.

